# Tamoxifen induces apoptosis through cancerous inhibitor of protein phosphatase 2A–dependent phospho-Akt inactivation in estrogen receptor–negative human breast cancer cells

**DOI:** 10.1186/s13058-014-0431-9

**Published:** 2014-09-17

**Authors:** Chun-Yu Liu, Man-Hsin Hung, Duen-Shian Wang, Pei-Yi Chu, Jung-Chen Su, Tsung-Han Teng, Chun-Teng Huang, Ting-Ting Chao, Cheng-Yi Wang, Chung-Wai Shiau, Ling-Ming Tseng, Kuen-Feng Chen

**Affiliations:** 1 Division of Hematology and Oncology, Department of Medicine, Taipei Veterans General Hospital, No. 201, Sec. 2, Shih-Pai Road, Taipei, 112 Taiwan; 2 School of Medicine, National Yang-Ming University, No. 155, Sec. 2, Li-Nong Street, Taipei, 112 Taiwan; 3 Department of Surgery, Taipei Veterans General Hospital, No. 201, Sec. 2, Shih-Pai Road, Taipei, 112 Taiwan; 4 Institute of Biopharmaceutical Sciences, National Yang-Ming University, No. 155 Sec. 2, Li-Nong Street, Taipei, 112 Taiwan; 5 Department of Pathology, St Martin De Porres Hospital, No. 565, Sec. 2, Daya Road, Chiayi, 600 Taiwan; 6 Department of Medical Research, National Taiwan University Hospital, No. 7, Chung-Shan South Road, Taipei, 100 Taiwan; 7 National Center of Excellence for Clinical Trial and Research, National Taiwan University Hospital, No. 7, Chung-Shan South Road, Taipei, 100 Taiwan; 8 Program in Molecular Medicine, School of Life Sciences, National Yang-Ming University, No. 155, Sec. 2, Li-Nong Street, Taipei, 112 Taiwan; 9 Department of Biological Science and Technology, National Chiao Tung University, No. 1001, University Road, Hsinchu, 300 Taiwan; 10 Department of Pathology, Taichung Hospital, Ministry of Health and Welfare, No. 199, Sec. 1, San-Min Road, Taichung, 403 Taiwan; 11 Division of Hematology & Oncology, Department of Medicine, Yang-Ming Branch of Taipei City Hospital, No. 105, Yusheng Street, Taipei, 112 Taiwan; 12 Medical Research Center, Cardinal Tien Hospital, School of Medicine, Fu Jen Catholic University, No. 362, Zhongzheng Road, New Taipei City, 231 Taiwan; 13 Department of Internal Medicine, Cardinal Tien Hospital, School of Medicine, Fu Jen Catholic University, No. 362, Zhongzheng Road, New Taipei City, 231 Taiwan

## Abstract

**Introduction:**

Tamoxifen, a selective estrogen receptor (ER) modulator, may affect cancer cell survival through mechanisms other than ER antagonism. In the present study, we tested the efficacy of tamoxifen in a panel of ER-negative breast cancer cell lines and examined the drug mechanism.

**Methods:**

In total, five ER-negative breast cancer cell lines (HCC-1937, MDA-MB-231, MDA-MB-468, MDA-MB-453 and SK-BR-3) were used for *in vitro* studies. Cellular apoptosis was examined by flow cytometry and Western blot analysis. Signal transduction pathways in cells were assessed by Western blot analysis. The *in vivo* efficacy of tamoxifen was tested in xenograft nude mice.

**Results:**

Tamoxifen induced significant apoptosis in MDA-MB-231, MDA-MB-468, MDA-MB-453 and SK-BR-3 cells, but not in HCC-1937 cells. Tamoxifen-induced apoptosis was associated with inhibition of cancerous inhibitor of protein phosphatase 2A (CIP2A) and phospho-Akt (p-Akt) in a dose-dependent manner. Ectopic expression of either CIP2A or Akt protected MDA-MB-231 cells from tamoxifen-induced apoptosis. In addition, tamoxifen increased protein phosphatase 2A (PP2A) activity, and tamoxifen-induced apoptosis was attenuated by the PP2A antagonist okadaic acid in the sensitive cell lines, but not in resistant HCC-1937 cells. Moreover, silencing CIP2A by small interfering RNA sensitized HCC-1937 cells to tamoxifen-induced apoptosis. Furthermore, tamoxifen regulated CIP2A protein expression by downregulating CIP2A mRNA. Importantly, tamoxifen inhibited the *in vivo* growth of MDA-MB-468 xenograft tumors in association with CIP2A downregulation, whereas tamoxifen had no significant effect on CIP2A expression and anti-tumor growth in HCC-1937 tumors.

**Conclusions:**

Inhibition of CIP2A determines the effects of tamoxifen-induced apoptosis in ER-negative breast cancer cells. Our data suggest a novel “off-target“ mechanism of tamoxifen and suggest that CIP2A/PP2A/p-Akt signaling may be a feasible anti-cancer pathway.

**Electronic supplementary material:**

The online version of this article (doi:10.1186/s13058-014-0431-9) contains supplementary material, which is available to authorized users.

## Introduction

Breast cancer, a major worldwide health threat, is considered to comprise a group of biologically heterogeneous diseases [1]-[3]. Breast cancer can be classified into different subgroups by the expression of estrogen receptor (ER), progesterone receptor (PR) and human epidermal growth factor receptor 2 (HER2). These subgroups present with distinct molecular backgrounds and exhibit diverse clinical behavior and treatment response [2],[4]. Among all breast cancers, tumors with negative expression of ER, which accounts for 25% to 30% of breast cancer [4],[5], is known for its aggressive nature and high metastatic potential [6]. Except for patients with the HER2-amplifying breast cancer subtype, the mainstay treatment for patients with ER-negative breast cancers is chemotherapy [7],[8]; however, clinical outcomes remain unsatisfactory [2]. Therefore, discovery of novel therapeutic approaches is needed to advance the treatment outcomes of patients with ER-negative breast cancers.

Protein phosphatase 2A (PP2A) has been shown to be an important tumor suppressor protein, and loss of PP2A function has been identified in several malignancies, such as lung, skin, colon, liver and breast cancers [9]-[11]. PP2A functions as a serine/threonine phosphatase and has been shown to regulate the activity of several oncogenic proteins, such as c-Myc, extracellular signal-regulated kinases and Akt, through direct dephosphorylation, [9],[12]-[14]. In breast cancer, PP2A has been shown to prevent the oncogenic transformation of human breast epithelial cells [13], and, conversely, mutant PP2A was not found to be able to suppress the oncogenic activity of RalA [15]. Recently, an emerging human oncoprotein called *cancerous inhibitor of PP2A* (CIP2A) has been shown to inhibit PP2A activity [16]. It is overexpressed in many cancers, including breast cancer [17]-[22]. Importantly, CIP2A overexpression is associated with clinical aggressiveness in human breast cancer and promotes the malignant growth of breast cancer cells [17]. Interestingly, the traditional chemotherapeutic agent doxorubicin has been shown to downregulate CIP2A expression, and increased CIP2A expression confers doxorubicin resistance in breast cancer cells [23]. Moreover, in our recent studies, we found that CIP2A is an important molecular determinant of bortezomib-induced apoptosis in leukemia cells [24] and in breast cancer cells [25]. Together, these data suggest that CIP2A has an important role in breast cancer cells and that targeting CIP2A could be a new therapeutic approach.

Tamoxifen, a selective estrogen-receptor modulator, is an important therapeutic agent for patients with ER-positive breast cancers [26]. The antiestrogenic activity of tamoxifen, by competing with estrogen for binding to the ER in tumor tissue, is considered to be its core mechanism of action, and adjuvant use of tamoxifen after primary resection of ER-positive breast tumor decreases the risk of recurrence [27]. Interestingly, in clinical trials, tamoxifen showed a 10% to 15% response rate in tumors without ER expression [26],[28]. Moreover, adjuvant tamoxifen treatment after excision of breast ductal carcinoma *in situ* (DCIS), a form of noninvasive breast carcinoma, has been shown to reduce recurrent risk, even in lesions without ER expression [8]. These clinical findings suggest that tamoxifen has certain ER-independent anticancer properties. Indeed, Blackwell *et al*. found that tamoxifen could inhibit angiogenesis in an ER-negative fibrosarcoma model [29]. Using the ER-negative HeLa cell model, Obrero *et al*. found that tamoxifen and its active metabolite could induce mitochondrial dysfunction and subsequently result in apoptotic cell death [30]. Using a virtual screening approach, Rongmin *et al*. found that 4-hydroxytamoxifen, the active metabolite of tamoxifen, could cause activation of Hsp90 ATPase at micromolar drug concentrations [31]. However, little has been explored regarding the hormone-independent effect of tamoxifen in breast cancer cells.

In this study, we report the apoptotic effect and mechanism of tamoxifen in ER-negative breast cancer cells. We found that downregulation of CIP2A and phospho-Akt (p-Akt) correlated with tamoxifen-induced apoptosis in ER-negative breast cancer cells. Moreover, overexpression of CIP2A or Akt reduced the apoptotic effects in tamoxifen-sensitive cells, whereas silencing CIP2A by small interfering RNA (siRNA) sensitized resistant cells to tamoxifen-induced apoptosis. Importantly, tamoxifen inhibited *in vivo* xenograft tumor growth in association with CIP2A downregulation. Therefore, tamoxifen induced apoptosis through downregulating CIP2A/PP2A/p-Akt signaling in ER-negative breast cancer cells.

## Methods

### Reagents and antibodies

Tamoxifen, okadaic acid and forskolin for *in vitro* experiments were purchased from Cayman Chemical (Ann Arbor, MI, USA). Tamoxifen citrate tablets obtained from AstraZeneca (London, UK) were used for *in vivo* animal experiments. For *in vitro* studies, tamoxifen at various concentrations was dissolved in dimethyl sulfoxide (DMSO) and added to cells in Dulbecco’s modified Eagle’s medium (Invitrogen, Carlsbad, CA, USA). The final DMSO concentration was 0.1% after addition to the medium. Antibodies for immunoblotting of CIP2A and ER-α, Ets1, Elk1 and lamin B were purchased from Santa Cruz Biotechnology (Santa Cruz, CA, USA) for immunoblotting. Other antibodies, such as Akt, p-Akt (Ser473), poly(ADP-ribose) polymerase (PARP) and Myc-tag, were obtained from Cell Signaling Technology (Danvers, MA, USA).

### Cell culture and Western blot analysis

The HCC-1937, MDA-MB-231, MDA-MB-468, MDA-MB-453, SK-BR-3 and MCF-7 cell lines were obtained from American Type Culture Collection (Manassas, VA, USA). All breast cancer cells were maintained in Dulbecco’s modified Eagle’s medium supplemented with 10% fetal bovine serum, 0.1 mM nonessential amino acids, 2 mM L-glutamine, 100 U/ml penicillin G, 100 μg/ml streptomycin sulfate and 25 μg/ml amphotericin B in a 37°C humidified incubator and an atmosphere of 5% CO_2_ in air. Lysates of breast cancer cells treated with drugs at the indicated concentrations for various periods of time were prepared for immunoblotting of p-Akt, Akt, *CIP2A* and other cells. Western blot analysis was performed as previously reported [25].

### Apoptosis analysis

Drug-induced apoptotic cell death was assessed using measurement of apoptotic cells by flow cytometry (sub-G1 analysis of propidium iodide’stained cells) and Western blot analysis of PARP cleavage.

### Gene knockdown using small interfering RNA

*SMART*pool siRNA reagents, including control (D-001810-01) and CIP2A, were all purchased from Dharmacon (Lafayette, CO, USA). The experimental procedure we carried out has been cpoibed previously [25]. Briefly, cells were transfected with siRNA (final concentration of 100 nM) in six-well plates using the liposome transfection reagent Lipofectamine 2000 (Lipo2000; Invitrogen) according to the manufacturer’s instructions. After 72 hours, the medium was replaced and the breast cancer cells were incubated with tamoxifen, harvested and separated for Western blot analysis and apoptosis analysis by flow cytometry.

### Generation of MDA-MB-231 cells with constitutively active Akt and MDA-MB-231 cells with constitutively active CIP2A

CIP2A cDNA (KIAA1524) was purchased from OriGene (RC219918; Rockville, MD, USA) and constructed into a pCMV6 vector. MDA-MB-231 cells were transfected with Myc-tagged Akt1 construct or Myc-tagged CIP2A as previously described [24]. Briefly, following transfection, cells were incubated in the presence of geneticin (G418, 1.40 mg/ml; Invitrogen). After 8 weeks of selection, surviving colonies (that is, those arising from stably transfected cells) were selected and individually amplified.

### Protein phosphatase 2A activity assay

The phosphatase activity of PP2A was detected by using a commercial PP2A immunoprecipitation phosphatase assay kit (EMD Millipore, Billerica, MA, USA) according to the manufacturer’s instructions and as previously described [24]. In brief, drug-treated or control cells were lysed, and PP2A was immunoprecipitated with anti-PP2A C subunit antibodies and protein A agarose beads overnight. Protein phosphatase activity of PP2A was determined by measuring the generation of free phosphate from threonine phosphopeptide using the malachite green phosphate complex assay. To avoid variability due to differences in the amounts of immunoprecipitated protein between samples, the phosphatase activities were normalized to the amount of PP2A immunoprecipitated, as detected and quantified by immunoblot analysis for each treatment group.

### Luciferase reporter constructs for the CIP2A promoter and 5′ detection analysis

The upstream region of the CIP2A promoter containing exon 1 (-2,000 bp to −1 bp) was amplified by PCR from the genomic DNA of cells and cloned into the reporter vector, Firefly vector (pGL4.17; Promega, Madison, WI, USA) by KpnI and BglII restriction sites. PCR-amplified promoter regions −2,000/−1, −400/−1, −110/−1 and −62/−1 were cloned into the KpnI and BglII restriction sites of the pGL4 basic vector. The nucleotide sequence of the clones was verified by sequencing.

### Dual-luciferase reporter assay

The promoter activity of *CIP2A* was determined using the dual-luciferase reporter assay kit (Promega) according to the manufacturer’s instructions. MDA-MB-468 cells were cotransfected with the luciferase reporter constructs pGL4.17-*CIP2A*-promoter (Firefly fluorescence reporter) and PGL4.74-Renilla (Renilla fluorescence reporter) as indicators for normalization of transfection efficiency. Twenty-four hours posttransfection, cells were treated with tamoxifen (5 μM) or DMSO for 24 hours. Cells were then lysed and assayed for luciferase activity. The promoter activity was repeated three times in parallel for statistical analysis.

### Chromatin immunoprecipitation assay

Chromatin immunoprecipitation (ChIP) was performed using the Pierce Agarose ChIP Kit (Pierce Biotechnology/Thermo Fisher Scientific, Rockford, IL, USA) according to the manufacturer’s instructions. Briefly, 1 × 10^7^ MDA-MB-468 cells were treated with tamoxifen 7.5 μM or DMSO for 24 hours. Physical cross-linking between chromatin (DNA) and proteins was fixed by 1% formaldehyde at room temperature for 10 minutes. Next, cells were lysed for DNA by enzyme digestion (micrococcal nuclease, 37°C, 15 minutes), and phosphatase inhibitor and protease inhibitor were added in the cell lysis step to avoid protein degradation. Lysates were clarified by centrifugation at 12,500 × *g* for 5 minutes at 4°C. Immunoprecipitation was performed by adding Elk1, Ets1 or rabbit immunoglobulin G antibodies as the negative control. The immunocomplex was precipitated by incubation with 25 μl of protein A/G magnetic beads for 1 hour at 4°C. The protein–DNA complex was eluted using 200 μl of elution buffer from the beads. Cross-linking of protein–DNA was reversed by adding 8 μl of 5 M NaCl at 95°C for 15 minutes. The DNA was purified using spin columns, and 2 μl of the DNA was used in the semi-PCR for amplification of the CIP2A promoter region (-139/-16 bp). Anti-RNA polymerase II antibody and glyceraldehyde 3-phosphate dehydrogenase (GAPDH) primers were provided by the manufacturer as a positive control for the assay technique and reagent integrity.

### Xenograft tumor growth

The animal study was approved by the Institutional Animal Care and Use Committee of Taipei Veterans General Hospital. All experimental procedures using these mice were performed in accordance with protocols approved by the Institutional Animal Care and Use Committee of Taipei Veterans General Hospital. Male NCr athymic nude mice (5 to 7 weeks of age) were obtained from the National Laboratory Animal Center (Taipei, Taiwan, Republic of China). The mice were housed in groups and maintained in a specific pathogen-free environment. Each mouse was inoculated subcutaneously in the dorsal flank while under isoflurane anesthesia with 5 × 10^6^ breast cancer cells suspended in 0.1 ml of serum-free medium containing 50% Matrigel (BD Biosciences, San Jose, CA, USA). Tumors were measured using calipers, and their volumes were calculated using a standard formula: Width^2^ × Length × 0.52. When tumors reached around 200 mM^3^, mice were orally administered 100 mg/kg body weight tamoxifen citrate three times weekly for 4 to 5 weeks. Controls received vehicle (1× phosphate-buffered saline). Mice were killed upon termination of treatment, and xenografted tumors were harvested and assayed for molecular events by Western blot analysis.

### RT-PCR

Total RNA was extracted from cultured cells using TRIzol reagent (Invitrogen), and RT-PCR was performed according to the manufacturer’s instructions (MBI Fermentas, Vilnius, Lithuania). RT-PCR analyses were performed as previously described [25] with specific primers for human *CIP2A* (forward primer, 5′-TGGCAAGATTGACCTGGGATTTGGA-3′; reverse primer, 5′-AGGAGTAATCAAACGvTGGGTCCTGA-3′; 172 bp), and the *GAPDH* gene was chosen as an internal control (forward primer, 5′-CGACCACTTTGTCAAGCTCA-3′; reverse primer, 5′-AGGGGTCTACAT GGCAACTG-3′; 228 bp). qRT-PCR was performed in a LightCycler 480 Instrument II (Roche Diagnostics, Indianapolis, IN, USA) using a LightCycler 480 SYBR Green I Master kit (Roche Diagnostics). The primers were the same as those above described.

### Immunohistochemical staining

Paraffin-embedded breast cancer tissue sections (4 μM) on poly-L-lysine-coated slides were deparaffinized and rinsed with 10 mM Tris-HCl (pH 7.4) and 150 mM sodium chloride. Peroxidase was quenched with methanol and 3% hydrogen peroxide. Slides were then placed in 10 mM citrate buffer (pH 6.0) at 100°C for 20 minutes in a pressurized heating chamber. After incubation with a 1:200 dilution of p-Akt1/2/3 (Thr 308)-R antibody (sc-16646-R; Santa Cruz Biotechnology) and with a 1:100 dilution of CIP2A antibody (ab84547; Abcam, Cambridge, UK) for 1 hour at room temperature, slides were thoroughly washed three times with phosphate-buffered saline. Bound antibodies were detected using the EnVision Detection Systems Peroxidase/DAB, Rabbit/Mouse kit (Dako, Glostrup, Denmark). The slides were then counterstained with hematoxylin. Paraffin-embedded sections of mouse kidney tissue and human colon carcinoma were used as positive controls for p-Akt1/2/3 and CIP2A, respectively, as described in the datasheet from the manufacturer. Negative controls had the primary antibody replaced by phosphate-buffered saline. A board-certified pathologist assessed the expression of p-Akt1/2/3 and CIP2A semiquantitatively based on the intensity of staining. The intensity of staining was scored as negative, weak, moderate and strong staining.

This study was approved by the ethics committee of the Institutional Review Board of Taipei Veterans General Hospital. All informed consents from sample donors were in accordance with the Declaration of Helsinki and were obtained at the time of donation.

### Statistical analysis

Data are expressed as mean ± SD or SE. Statistical comparisons were based on nonparametric tests, and statistical significance was defined as *P* < 0.05. For survival analysis, progression-free survival curves of patients were generated using the Kaplan-Meier method and compared by performing a logrank test. All statistical analyses were carried out using SPSS for Windows software, version 12.0 (SPSS, Chicago, IL, USA).

## Results

### Differential apoptotic effects of tamoxifen on estrogen receptor–negative breast cancer cells

**Figure 1:**
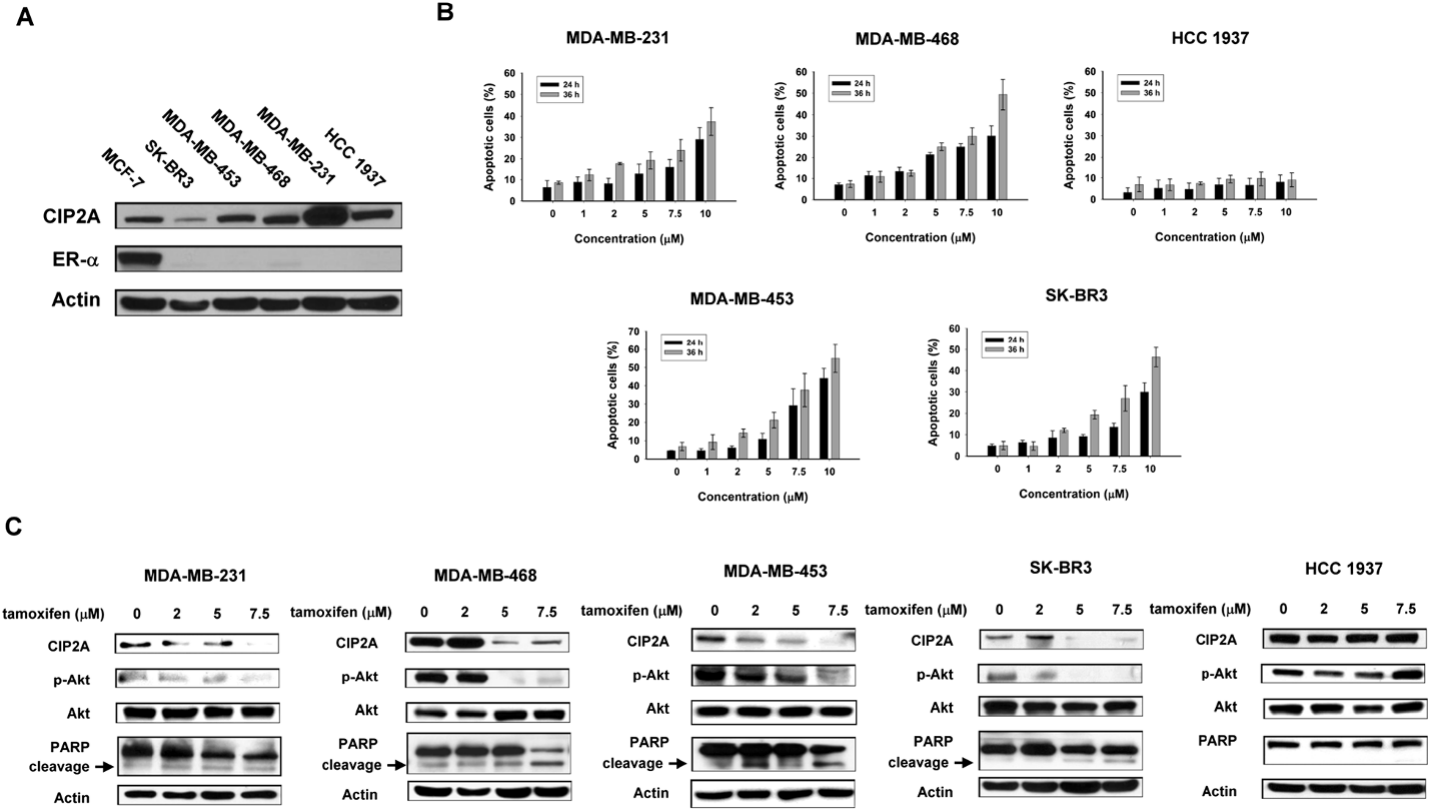
**Tamoxifen-induced apoptosis in association with downregulation of CIP2A and p-Akt in estrogen receptor–negative breast cancer cells. (A)** Differential cancerous inhibitor of protein phosphatase 2A (CIP2A) expression in five estrogen receptor (ER)–negative breast cancer cell lines (SK-BR3, MDA-MB-453, MDA-MB-468, MDA-MB-231 and HCC-1937). MCF-7 was used as a positive control for ER expression. **(B)** Dose and time escalation effects of tamoxifen on apoptosis in five ER-negative breast cancer cell lines. Cells were exposed to tamoxifen at the indicated doses (1 μM, 2 μM, 5 μM, 7.5 μM and 10 μM) for 24 and 36 hours. Apoptotic cells were determined by flow cytometry (sub-G_1_ analysis of propidium iodide–stained cells). Columns, Mean (*n* = 3); bars, SD. **(C)** Dose-dependent analysis of CIP2A, p-Akt and cleaved poly(ADP-ribose) polymerase (PARP). Cells were exposed to tamoxifen at the indicated doses for 36 hours. Cell lysates were prepared and assayed for these molecules by Western blotting. Data are representative of three independent experiments. Note that there was no significant tamoxifen-induced apoptosis in HCC-1937 cells; only the PARP preform is presented.

To understand the antitumor effect of tamoxifen on ER-negative breast cancer cells, we first assessed its apoptotic effect in a panel of five ER-negative human breast cancer cell lines: SK-BR3, MDA-MB-453, MDA-MB-468, MDA-MB-231 and HCC-1937. First, negative expression of ER in the cell lines was confirmed by Western blotting (Figure 1A). Flow cytometric analysis of sub-G_1_ cells showed that tamoxifen induced differential apoptotic effects at the indicated times (24 and 36 hours) and doses (1, 2, 5, 7.5 and 10 μM) in the five breast cancer cell lines (Figure 1B). Tamoxifen induced apoptosis in a dose- and time-dependent manner in MDA-MB-231, MDA-MB-468, MDA-MB-453 and SK-BR3 cells, whereas no apparent apoptotic effects were observed in HCC-1937 cells after tamoxifen treatment for 24 and 36 hours at doses up to 10 μM (Figure 1B). These results suggest that ER-negative breast cancer cell lines MDA-MB-231, MDA-MB-468, MDA-MB-453 and SK-BR3 cells are sensitive to the cytotoxic effect of tamoxifen, but that the HCC-1937 cell line is not.

### Tamoxifen induces apoptosis in association with downregulation of CIP2A and p-Akt in sensitive estrogen receptor–negative breast cancer cells

We previously found that bortezomib induced apoptosis in triple-negative (negative expression of ER, PR and HER2) breast cancer cells through downregulation of CIP2A and p-Akt, suggesting that CIP2A is a target of bortezomib [25]. In this study, we investigated the molecular events associated with apoptosis induced by tamoxifen in ER-negative breast cancer cells, with a particular focus on CIP2A. As revealed in Figure 1C, in the four tamoxifen-sensitive cell lines MDA-MB-231, MDA-MB-468, MDA-MB-453 and SK-BR3, the protein levels of CIP2A were downregulated by tamoxifen in a dose-dependent manner. Moreover, inhibition of CIP2A was associated with downregulation of p-Akt and induction of apoptosis (evident by increased cleavage of PARP). In contrast, the protein levels of CIP2A, p-Akt and PARP were not significantly affected by tamoxifen in HCC-1937 cells. The results suggest that inhibition of CIP2A may play a major role in tamoxifen-induced apoptosis in ER-negative breast cancer cells. We also tested the effects of tamoxifen on CIP2A and p-Akt in ER-positive MCF-7 cells (Additional file 1: Figure S1). As shown in Additional file 1: Figure S1, tamoxifen also induced apoptosis in association with CIP2A and p-Akt downregulation in MCF-7 cells.

Tamoxifen has a well-established ERα-selective partial agonist/antagonist function and can induce apoptotic as well as antiproliferative effects, which raises the issue whether ERα plays a distinct role in tamoxifen-induced effects on CIP2A, p-Akt and apoptosis. In this regard, we used fulvestrant (formerly ICI 182,780), a structural analogue of estrogen with a pure antagonist function on ERα, and tested the effects of fulvestrant in ERα-positive MCF-7 cells and ERα-negative MDA-MB-453 cells. To show the effect of fulvestrant on apoptosis in both ERα-positive cells and ERα-negative cells, we used a relative higher dose (1 μM) than the normally used doses (around 100 nM) for *in vitro* studies of ERα-positive cells [32]. As expected, both tamoxifen and fulvestrant could downregulate ERα in MCF-7 cells, a known effect contributing to apoptosis (Additional file 1: Figure S2). In addition, the CIP2A downregulation seemed more prominent in MDA-MB-453 cells than in MCF-7 cells; however, tamoxifen-induced apoptosis seemed higher in MCF-7 cells. Together our data show an “off-ERα” effect of tamoxifen. Because a second estrogen receptor, ERα, has been found to be expressed in 50% to 90% of ERα-negative breast cancers [33], we also checked the expression of ERα in MCF-7 cells and ERα-negative cells and found that these cells also showed positive ERα expression (Additional file 1: Figure S3).

### Target validation of the CIP2A/PP2A/p-Akt pathway as a molecular determinant of tamoxifen-induced estrogen receptor–negative breast cancer apoptosis

**Figure 2:**
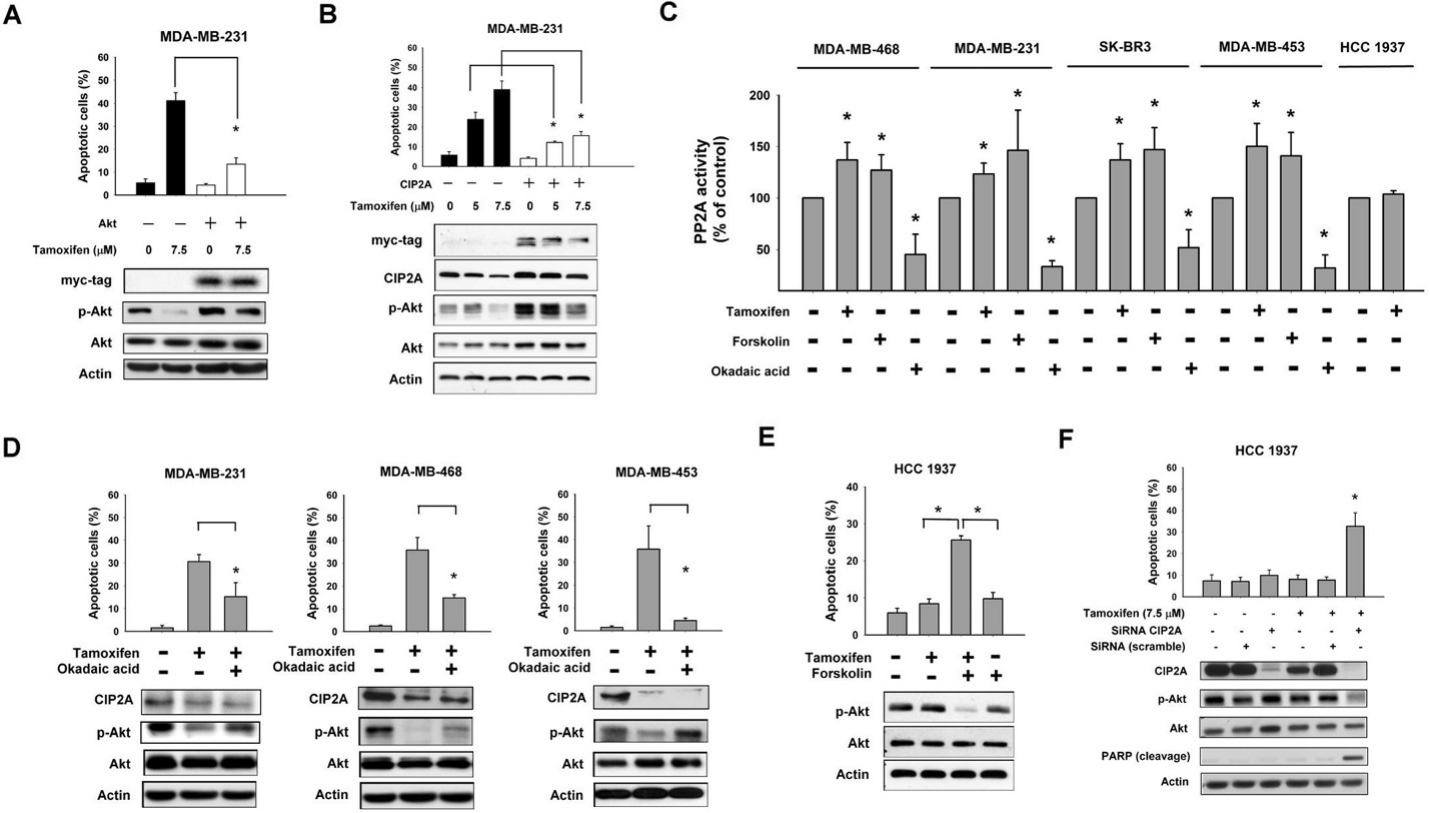
**CIP2A/PP2A/p-Akt mediated tamoxifen-induced apoptosis.** **(A)** Ectopicexpression of myc-tagged Akt protected MDA-MB-231 cells from tamoxifen-induced apoptosis. **(B)** Ectopic expression of myc-tagged CIP2A protected MDA-MB-231cells from tamoxifen-induced apoptosis. Note that cells with ectopic expression of myc-tagged CIP2A also had constitutively high p-Akt. For experiments **(A)** and **(B)**, cells were transfected as described in Material and Methods. Control cells were empty-vector cells. Apoptosis was analyzed by flow cytometry after cells were sequentially exposed to DMSO or tamoxifen at the indicated doses for 36 hours. **(C)** Analysis of PP2A activity in drug-treated cells. Cells were treated with DMSO ortamoxifen at 7.5 μM or okadaic acid at 20 nM (as a negative control) or forskolin 40μM (as a positive control) for 36 hours. Cell lysates were assayed for PP2A activity. **(D)** Pretreatment of PP2A inhibitor okadaic acid protected cells from tamoxifen-induced apoptosis. Cells were treated with DMSO (control) or tamoxifen (7.5 μM) for 36 hours. For pretreatment, cells were pretreated with okadaic acid(20nM) for 1 hour; then they were washed and treated with tamoxifen (7.5 μM) for 36hours. Cell lysates were separated and assayed for sub-G1 analysis and westernblotting. **(E)** Cotreatment of tamoxifen with forskolin enhanced apoptosis in resistant HCC-1937 cells. Cells were treated with DMSO (control), tamoxifen (7.5 μM), or co-treated with tamoxifen (7.5 μM) and forskolin (40 μM) for 36 hours. Cell lysates were separated and assayed for sub-G1 analysis and western blotting. **(F)** Downregulation of CIP2A by siRNA increased tamoxifen-induced apoptosis in HCC-1937 cells. Cells were transfected with either control (scrambled siRNA) or CIP2A siRNA for 72 hours followed by exposure to tamoxifen at 7.5 μM for 36 hours. For **(A)** to **(F)**, *Columns*, mean (n = 3); *bars*, SD; *P< 0.05.

To examine the role of CIP2A and p-Akt in mediating tamoxifen-induced apoptosis, we generated MDA-MB-231 cells with constitutive, ectopic expression of Myc-tagged Akt (Figure 2A) or Myc-tagged CIP2A (Figure 2B). Of note, MDA-MB-231 cells with constitutive, ectopic expression of Myc-tagged Akt also expressed constitutively activated p-Akt (Figure 2A). As shown in Figures 2A and 2B, constitutive ectopic expression of either Myc-tagged Akt or CIP2A protected sensitive MDA-MB-231 cells from apoptotic death induced by tamoxifen. Because CIP2A is a cellular inhibitor of PP2A [16],[24], we examined the PP2A activity in tamoxifen-treated cells. As shown in Figure 2C, tamoxifen significantly increased the phosphatase activity of PP2A in tamoxifen-sensitive cell lines (Figure 2C). In addition, okadaic acid, a PP2A inhibitor acting as a negative control, decreased the phosphatase activity of PP2A in these four cell lines; however, forskolin, a PP2A agonist acting as a positive control, increased PP2A activity in these cells (Figure 2C). Moreover, pretreatment with okadaic acid reduced the effects of tamoxifen on apoptosis and p-Akt in tamoxifen-sensitive MDA-MB-231, MDA-MB-468 and MDA-MB-453 cells (Figure 2D), whereas cotreatment with forskolin sensitized HCC-1937 cells to tamoxifen-induced apoptosis and p-Akt downregulation (Figure 2E). We also checked PP2A activity in these cells (Additional file 1: Figure S4). As shown in the Additional file 1: Figure S4, forskolin increased PP2A activity in HCC-1937 cells, and the combination of tamoxifen and forskolin further increased PP2A activity. Next, we performed knockdown of expression of CIP2A by using siRNA and found that CIP2A siRNA sensitized the resistant HCC-1937 cells to tamoxifen-induced apoptosis (Figure 2F). Notably, depletion of CIP2A alone did not induce significant apoptosis in tamoxifen-resistant HCC-1937 cells (Figure 2F); therefore, we further examined whether CIP2A siRNA alone induced apoptosis in the tamoxifen-sensitive MDA-MB-468 cells (Additional file 1: Figure S5). Similarly, CIP2A siRNA alone did not induce significant apoptosis. However, tamoxifen plus CIP2A siRNA induced significantly more apoptosis as compared to tamoxifen treatment (Additional file 1: Figure S5). It can be argued that CIP2A downregulation *per se* may also participate in the apoptosis mechanism. In this regard, we further tested the effects of common chemotherapeutic agents for breast cancers, including 5-fluorouracil (5-FU), paclitaxel and docetaxel, and compared them with tamoxifen in MDA-MB-468 cells (Additional file 1: Figure S6). We found that only tamoxifen induced significant CIP2A reduction, whereas all of these agents induced apoptosis in MDA-MB-468 cells. Together, these results indicate that the CIP2A/PP2A/p-Akt pathway plays a critical role in mediating the apoptotic effect of tamoxifen in ER-negative breast cancer cells.

Researchers in some studies have proposed other ER-independent therapeutic potential of tamoxifen, such as activation of Hsp90 [31]. Therefore, we performed coimmunoprecipitation experiments with CIP2A and Hsp90 in tamoxifen-treated MDA-MB-468 cells, and we found no apparent interactions between these two molecules upon tamoxifen treatment (Additional file 1: Figure S7). In addition, because PP2A comprises a large family of protein phosphatases known to affect apoptosis by regulating multiple pro- or antiapoptotic proteins, such as c-Myc and Bcl-2 [12]. It is possible that tamoxifen-induced CIP2A inhibition could also affect cell survival through dysregulation of PP2A substrates involved in apoptosis. Accordingly, we checked the effects of tamoxifen on c-Myc and Bcl-2 in tamoxifen-sensitive cells. Tamoxifen showed differential effects on c-Myc and Bcl-2 in sensitive cell lines (MDA-MB-468, MDA-MB-231, MDA-MD-453 and SK-BR3); it reduced c-Myc expression more prominently in SK-BR3 cells; and it reduced Bcl-2 expression more prominently in MDA-MB-231 cells (Additional file 1: Figure S8).

### Tamoxifen downregulates transcription of CIP2A in estrogen receptor–negative breast cancer cells

**Figure 3:**
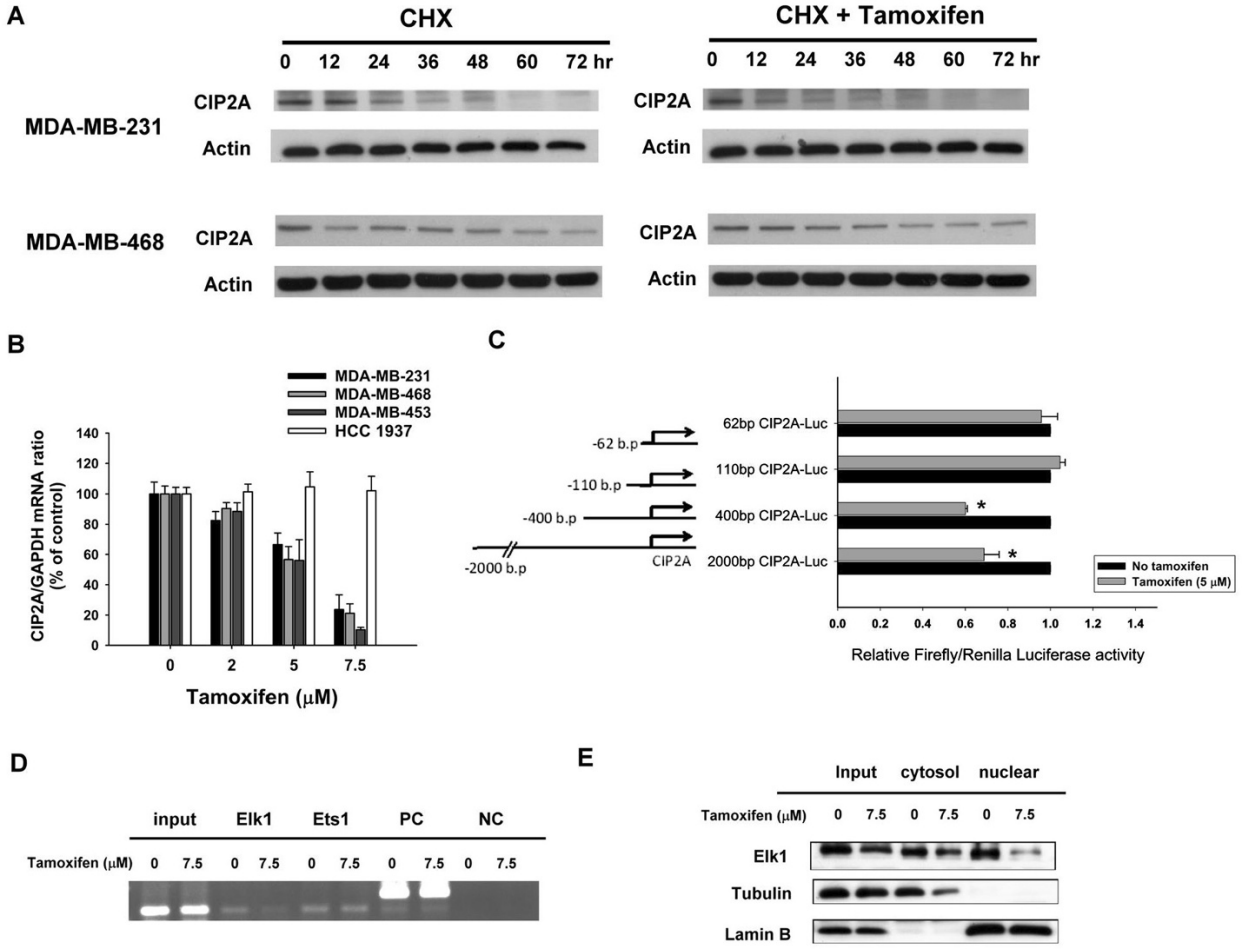
**Tamoxifen downregulated transcription of CIP2A.** **(A)** After cells were treated with 100 *μ*g/ml translation inhibitor cycloheximide (CHX) in the presence (left) or absence (right) of tamoxifen (7.5 *μ*M) for the indicated periods, the stability of CIP2A protein in whole-cell lysates was assessed by Western blot analysis. In tamoxifen-sensitive cells, the addition of tamoxifen did not affect CIP2A degradation. **(B)** Tamoxifen affects CIP2A transcription. Cells were treated with tamoxifen at the indicated doses for 36 hours, after which total RNA was isolated and CIP2A mRNA was assayed by quantitative RT-PCR. Columns, mean values (n = 3); bars, SD. GAPDH, glyceraldehyde 3-phosphate dehydrogenase. **(C)** Identification of *CIP2A* proximal promoter regions that were affected by tamoxifen treatment. MDA-MB-468 cells were transfected for 24 hours with Firefly luciferase reporter vectors carrying *CIP2A* promoters of different lengths and Renilla vectors and then treated for 24 hours with 5 *μ*M tamoxifen or DMSO. Cell lysates were then assayed for dual-luciferase activity as described in the Methods section. Columns, mean values (n = 3); bars, SD; *P < 0.05. **(D)** Tamoxifen disturbed binding of Elk1 to the *CIP2A* promoter region. Chromatin immunoprecipitation assays of the *CIP2A* promoter were performed as described in the Methods section. Soluble chromatin was immunoprecipitated with Elk1, Ets1 or immunoglobulin G (negative control (NC)) antibodies. Immunoprecipitates were subjected to PCR with primer pairs specific to *CIP2A* promoter (-16 to -139 bp). The gel shown is representative of three independent experiments. Anti-RNA polymerase II antibody and GAPDH primers were used as a positive control (PC). **(E)** Tamoxifen affected Elk1 expression. Nuclear and cytoplasmic extracts were prepared from MDA-MB-468 cells treated with tamoxifen (7.5 *μ*M) or DMSO for 24 hours. Cell lysates were examined by Western blotting for Elk1. Lamin B and tubulin were used as loading controls.

To further study how tamoxifen affected CIP2A expression, we first examined whether tamoxifen could affect CIP2A elimination (degradation) when translation was blocked by the protein synthesis inhibitor cycloheximide. Our data showed that after protein translation was blocked by cycloheximide, the rate of CIP2A degradation did not change significantly with or without tamoxifen treatment in MDA-MB-231 and MDA-MB-468 cells (Figure 3A). This implies that the effect of tamoxifen on CIP2A may occur at the pretranslation level. We next investigated whether tamoxifen affected CIP2A transcription via quantitative RT-PCR analysis. As shown in Figure 3B, CIP2A mRNA levels decreased in a dose-dependent manner upon treatment with tamoxifen in sensitive MDA-MB-231, MDA-MB-468 and MDA-MB-453 cells, but not in resistant HCC-1937 cells (Figure 3B). These results indicate that tamoxifen inhibited CIP2A transcription, and failure of this inhibition suggests tamoxifen resistance in ER-negative breast cancer cells.

To further decipher the possible mechanism how tamoxifen reduced CIP2A mRNA, we presumed that tamoxifen may affect CIP2A promoter activity through transcription factors because researchers in previous studies have unraveled several transcriptional regulators of CIP2A promoter [34],[35]. Accordingly, MDA-MB-468 cells were transfected with luciferase reporter constructs for CIP2A promoter of varying lengths (Figure 3C). As shown in Figure 3C, tamoxifen significantly downregulated the activity of CIP2A promoter in cells transfected with constructs of −1 to approximately −2,000 bp and −1 to about −400 bp, but tamoxifen did not reduce CIP2A promoter activity in cells transfected with constructs of −1 to approximately −110 and −1 to about −62 bp. According to previous studies [34],[35], Ets1 and Elk1 could bind to promoter regions between −400 and −110 bp. Next, we performed ChIP assays (Figure 3D) to examine whether the binding of Ets1 or Elk1 (or both) to CIP2A promoter is affected by tamoxifen. We found that tamoxifen disturbed the binding of Elk1 to CIP2A promoter. Further Western blot analysis for Elk1 in nuclear/cytoplasmic extracts from MDA-MB-468 cells treated with or without tamoxifen revealed that tamoxifen reduced Elk1 expression in the nuclear extracts (Figure 3D). These data suggest that tamoxifen may downregulate CIP2A transcription by affecting Elk1.

### Effect of tamoxifen on estrogen receptor–negative breast cancer xenograft tumor growth in vivo

**Figure 4:**
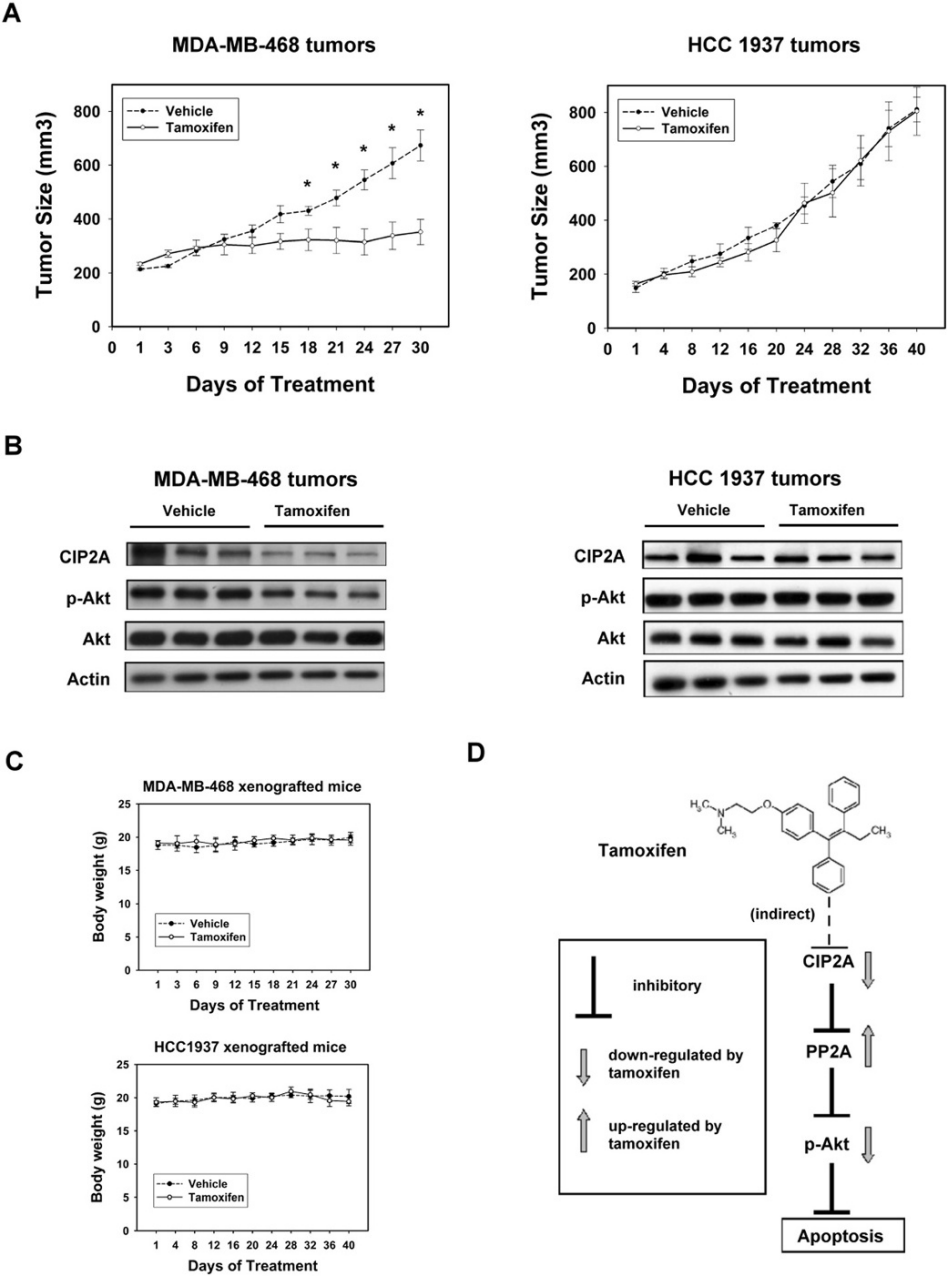
***In vivo*** **effect of tamoxifen on human breast cancer cell lines in xenograft nude mice. (A)** Tamoxifen treatment decreased the size of MDA-MB-468 tumors (left) but did not affect HCC-1937 tumor cell growth (right). Points, mean values (*n* = 6); bars, SE; *P < 0.05. Mice received either 100 mg/kg body weight tamoxifen citrate administered orally three times weekly or vehicle (1× phosphate-buffered saline), as described in the Methods section. **(B)** Western blot analysis of the expression levels of cancerous inhibitor of protein phosphatase 2A (CIP2A), phospho-Akt (p-Akt) and Akt in MDA-MB-468 and HCC-1937 xenografts treated with control or tamoxifen. **(C)** Body weight of xenograft mice bearing MDA-MB-468 (top) and HCC-1937 (bottom) tumors. Points, mean values (*n* = 6); bars, SD. **(D)** Schema of the molecular mechanism of the action of tamoxifen on the CIP2A/PP2A pathway. By inhibiting CIP2A, tamoxifen restores protein phosphatase 2A (PP2A) activity downregulating p-Akt and leading to subsequent cell apoptosis.

To confirm that using tamoxifen to inhibit CIP2A has potentially clinically relevant implications in ER-negative breast cancer, we next used ER-negative breast cancer xenograft models to evaluate the effect of tamoxifen *in vivo*. Mice with MDA-MB-468- and HCC-1937-xenografted tumors were generated to validate the role of CIP2A *in vivo*. After successfully establishing the xenograft model in nude mice, these tumor-bearing mice were treated with tamoxifen at the dose of 100 mg/kg or vehicle (as control) orally three times per week for 4 to 5 weeks. As shown in Figure 4A, tamoxifen inhibited MDA-MB-468 tumor growth significantly, whereas HCC-1937 tumor growth was not affected. Furthermore, the protein expression of CIP2A, p-Akt and Akt were checked to confirm the correlation between the biological response observed *in vivo* and the molecular mechanism discovered *in vitro* (Figure 4B). Tamoxifen inhibited the expression of CIP2A and p-Akt consistently in the three representative MDA-MB-468 tumors, whereas no significant change was seen in the control (vehicle-treated) tumors (Figure 4B, left panel). In the HCC-1937 tumors, the expression of CIP2A and p-Akt were not affected in either the tamoxifen- or control-treated tumors (Figure 4B, right panel). At the end of the experiment, all the animals had tolerated the treatments quite well without observable signs of toxicity and had stable body weights throughout the whole treatment course (Figure 4C). A schema summarizing the molecular mechanism of tamoxifen in sensitive ER-negative breast cancer cells is presented in Figure 4D. Tamoxifen inhibited CIP2A, restored PP2A activity and led to p-Akt downregulation and cancer cell apoptosis (Figure 4D).

### Expression of CIP2A correlates with expression of p-Akt in breast tumor tissue from patients with estrogen receptor–negative breast cancers

**Figure 5:**
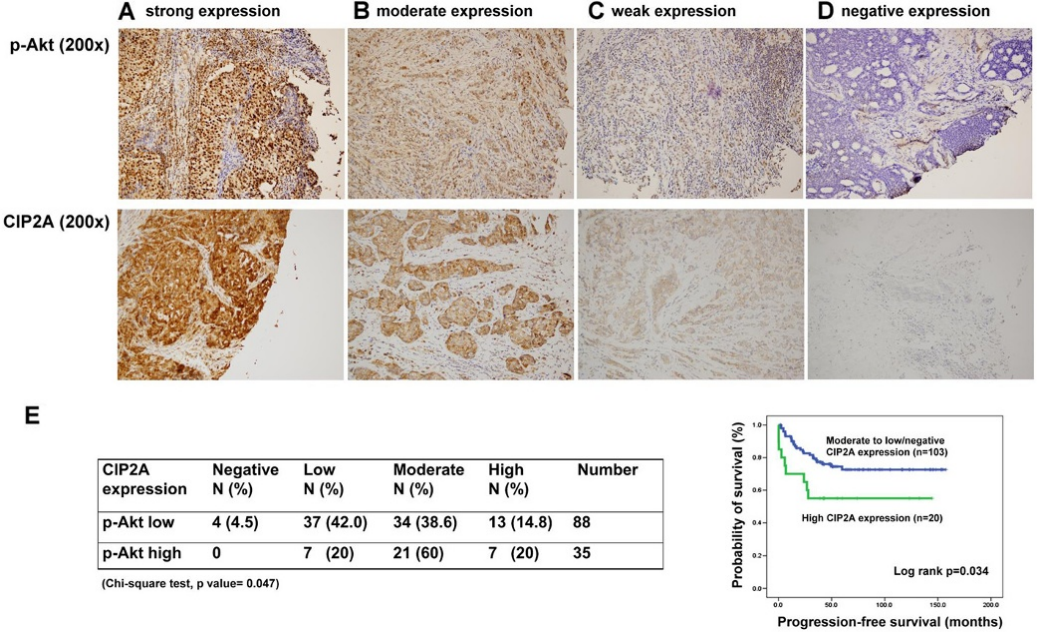
**Immunohistochemical intensity of nuclear expression for p-Akt correlates with cytoplasmic staining for CIP2A.** Upper panel shows representative immunohistochemical patterns of p-Akt in estrogen receptor (ER)–negative human breast cancer tissues showing strong nuclear expression **(A)**, moderate nuclear expression **(B)**, mild nuclear expression **(C)** and negative nuclear expression **(D)**. No cytoplasmic expression of p-Akt was noted. Lower panel shows representative immunohistochemical patterns of cancerous inhibitor of protein phosphatase 2A (CIP2A) in cancer cells showing strong cytoplasmic expression **(A)**, moderate cytoplasmic expression **(B)**, mild cytoplasmic expression **(C)**, negative cytoplasmic expression **(D)**. Original magnification in upper and lower panels, 200×. **(E)** Left: CIP2A and p-Akt expression in tumor samples from 123 patients with ER-negative breast cancers (see Tables 1 and 2 for details). Right: Progression-free survival curves for patients with high CIP2A expression (*n* = 20) and patients with moderate to low or negative CIP2A expression (*n* = 103). Curves were generated by using the Kaplan-Meier method and compared by performing a logrank test.

In four representative breast tumor tissues from patients with ER-negative breast cancers with varying degrees (negative, weak, moderate and strong staining) of CIP2A expression, immunohistochemical staining for p-Akt showed that the intensity of nuclear expression for p-Akt correlated with cytoplasmic staining for CIP2A (Figures 5A to 5D). Our previous data showed that 50 (87.7%) of 57 tumor samples from triple-negative breast cancer patients demonstrated variable CIP2A expression levels [25]. A recent immunohistochemistry-based study demonstrated that CIP2A signature clustered with basal-type and HER2-positive breast cancer signatures and suggested that CIP2A is linked to these two subtypes of breast cancer [36]. To study the clinical significance of CIP2A, we further examined CIP2A and p-Akt expression in tumor samples from 123 patients with ER-negative breast cancers (including 53 (43.1%) with HER2-positive breast cancers). As shown in Table 1, 96.7% of patients had variable CIP2A expression levels (from low to high). High expression of CIP2A was significantly correlated with the patient’s clinical stage at diagnosis, as well as with pathological microvascular invasion, but it was not significantly associated with age or HER2 status (Table 2). Survival analysis showed that patients with high CIP2A expression had worse progression-free survival as compared with patients with moderate to low or negative CIP2A expression (Table 2, Figure 5E). Moreover, moderate to high CIP2A expression correlated with high p-Akt expression in these tumor samples (Figure 5E). The *in vivo* result, therefore, supported the correlation of CIP2A and p-Akt signaling found *in vitro*.

**Table 1:** **General characteristics of primary tumor samples from 123 patients with hormone receptor–negative breast cancers** ^**a**^

Characteristic	***N*** = 123	%
Median age, yr (IQR)	54 (48 to 66)	
Tumor stage	32	26.0
T1	79	64.2
T2	7	5.7
T3	4	3.3
T4	1	0.8
Node-positive disease	57	47.3
Metastatic disease	6	4.9
TNM stage		
I	19	15.4
II	70	56.9
III	28	22.8
IV	6	4.9
HER2-positive^b^	53	43.1
CIP2A expression		
Negative	4	3.3
Low	44	35.8
Moderate	55	44.7
High	20	16.3
Positive p-Akt expression	120	97.6

^a^CIP2A, Cancerous inhibitor of protein phosphatase 2A HER2, Human epidermal growth factor receptor 2. ^b^HER2-positive was defined as an immunohistochemical staining score of 3+ or 2+ with gene amplification shown by fluorescence *in situ* hybridization.

**Table 2:** **Clinicopathological characteristics according to CIP2A expression** ^**a**^

Characteristic	Moderate to low CIP2A expression,	High CIP2A expression,	***P***-value
	***n*** = 103 (%)	***n*** = 20 (%)	
Median age (IQR)	55 (49.0 to 67.0)	49 (40.75 to 55.50)	0.401
Advanced clinical stage (stages III and IV)	23 (22.3)	11 (55.0)	0.011
Microvascular invasion	23 (22.3)	9 (45.0)	0.001
HER2-positive^b^	47 (45.6)	6 (30.0)	0.337
High p-Akt expression	49 (47.6)	12 (60.0)	0.338
5-year PFS^c^	76.7%	55%	0.034

^a^CIP2A, Cancerous inhibitor of protein phosphatase 2A; HER2, Human epidermal growth factor receptor 2; PFS, Progression-free survival. ^b^HER2-positive was defined as an immunohistochemical staining score of 3+ or 2+ with gene amplification shown by fluorescence *in situ* hybridization. ^c^Median follow-up period 50.5 months (IQR = 34.1 to 86.8).

## Discussion

In this study, we demonstrate the mechanism of action of the drug tamoxifen in ER-negative breast cancer cells, that is, induction of cancer cell apoptosis through CIP2A-dependent p-Akt downregulation. These findings not only increase current understanding of the drug mechanisms of tamoxifen but also support the rationale of targeting CIP2A in future drug development for ER-negative breast cancer.

Our data clearly show an “off-ERα” effect of tamoxifen (Figure 1 and Additional file 1: Figures S1 and S2). In addition, we checked the expression of ERα in MCF-7 and ERα-negative cells (Additional file 1: Figure S3). Indeed, these cells showed positive ERα expression. ERα, a second estrogen receptor, has been found to be expressed in 50% to 90% of ERα-negative breast cancers [33]. Although tamoxifen is believed to target ERα in ER-positive breast cancers, this mixed agonist-antagonist can also transactivate ERα, raising the question whether ERα has a prognostic value for tamoxifen responsiveness/resistance [37]. Clinical studies have shown ERα expression to be a good prognostic marker for tamoxifen responsiveness in both ER-positive and ER-negative breast cancer patients [33],[37]-[39]. *In vitro* studies have suggested a tumor-suppressive role of ERα, particularly in ER-positive breast cancer cells [33],[37]. Whether ERα has multiple distinct roles in ERα-negative breast cancers needs further investigation. It would be interesting to see whether ERα plays a role in tamoxifen-induced effects on CIP2A/PP2A/p-Akt mechanisms, which requires more studies.

There is a growing body of evidence supporting the use of CIP2A as an anticancer target [16]-[20],[23]-[25],[40]-[43]. Accordingly, several agents that inhibit CIP2A have been identified, and some have demonstrated efficacy against different cancer cells. In our previous studies, we demonstrated that bortezomib, a proteasome inhibitor, induced apoptosis via proteasome-independent inhibition of CIP2A in triple-negative breast cancer cells [25], hepatocellular carcinoma cells [44], leukemia cells [24] and head and neck cancer cells [41]. Interestingly, erlotinib, as well as its derivatives, could also promote cancer cell death by affecting the same signaling pathway (CIP2A/PP2A/p-Akt) [42],[45]. In this study, we found that tamoxifen, a selective estrogen antagonist, induced significant cancer cell apoptosis in ER-negative breast cancer cells (Figure 1B). We confirmed the role of the CIP2A/PP2A/p-Akt signaling pathway in tamoxifen-induced apoptosis in ER-negative breast cancer cells. Liang *et al*. found that rabdocoetsin B, an herbal extract of *Rabdosia coetsa*, also inhibited proliferation and induced apoptosis in lung cancer cells through CIP2A-dependent p-Akt downregulation [43]. Recently, Jung *et al.* found that a tumor-suppressive microRNA, miR-375, could suppress CIP2A and CIP2A-dependent Myc protein levels in oral cancer cells that resulted in inhibition of cancer cell proliferation, migration and invasion [46]. Furthermore, Sung *et al.* showed that when expression of CIP2A in tumor cells was induced by IL-10, the aggressiveness of pulmonary adenocarcinoma was aggravated [47]. We conclude from current evidence that CIP2A is an important molecule associated with cancer cell survival and could be a potential anticancer target in many malignant diseases.

It is noteworthy, however, that depletion of CIP2A alone did not induce significant apoptosis in tamoxifen-resistant HCC-1937 cells (Figure 2F). Similarly, CIP2A siRNA alone did not induce significant apoptosis in tamoxifen-sensitive MDA-MB-468 cells (Additional file 1: Figure S5). In a previous study, Come *et al*. [17] showed that CIP2A siRNA alone decreased the proliferation of MDA-MB-231 cells and inhibited the growth of xenograft MDA-MB-231 cells *in vivo*. However, Junttila *et al*. [16] and Côme *et al*. [17] showed that CIP2A siRNA alone does not induce significant apoptosis, but significantly inhibits cell proliferation. Consistent with previous studies, our data show depletion CIP2A *per se* is insufficient to induce apoptosis, but plays a key role in mediating apoptosis induced by several “CIP2A-ablating agents,” including tamoxifen [25],[42],[44],[45],[48]. These results suggest CIP2A is essential but not sufficient in mediating tamoxifen-induced apoptosis. Alternatively, it can be argued that CIP2A downregulation *per se* may also participate in the apoptosis mechanism. We further tested the effects of common chemotherapeutic agents for breast cancers, including 5-FU, paclitaxel and docetaxel, compared with tamoxifen in MDA-MB-468 cells (Additional file 1: Figure S6). Interestingly, only tamoxifen induced significant CIP2A reduction, whereas all these agents induced apoptosis in MDA-MB-468 cells. More studies are needed to elucidate whether and at which steps CIP2A downregulation participates in the process of apoptosis.

Although most clinical evidence suggests that tamoxifen is particularly effective for ER-positive breast cancer because it acts by competitive inhibition of ER and estrogen-regulated genes, which slows tumor progression [26],[49], a 10% to 15% response rate has been reported in patients with ER-negative breast cancer [26]. Tamoxifen has also been shown to provide some protection in patients with ER-negative DCIS after resection [50],[51]. Researchers in some other studies have proposed the ER-independent therapeutic potential of tamoxifen, including in antiangiogenesis [29], induction of mitochondrial dysfunction [30] and activation of Hsp90 [31], but none of these studies were conducted in breast cancer cells. Moreover, we performed coimmunoprecipitation experiments with CIP2A and Hsp90 in tamoxifen-treated MDA-MB-468 cells and found no apparent interactions between these two molecules upon tamoxifen treatment (Additional file 1: Figure S7). In contrast with previous studies, in our present study we explored the mechanism of tamoxifen using ER-negative breast cancer cell lines and identified a new mechanism of action of tamoxifen, namely, CIP2A-dependent p-Akt inhibition. Our results may partly explain why some ER-negative breast cancer patients respond to tamoxifen [8],[26],[28]. Future studies correlating response to tamoxifen with downregulation and/or pretreatment expression levels of CIP2A in breast cancer patients may help to establish a clinical role for CIP2A as a predictive factor in breast cancer. Furthermore, in our previous studies, we showed that bortezomib, by inhibiting the CIP2A/PP2A/p-Akt pathway, could sensitize hepatocellular carcinoma cells to an antihuman death receptor 5 antibody, CS-1008 [48], and could enhance radiation-induced apoptosis in cervical cancer and hepatocellular carcinoma cells [52]. Therefore, the potential application of the new CIP2A inhibitory mechanism of tamoxifen as part of a combination treatment strategy with other anti–breast cancer agents is worth further investigation.

In addition, we checked the effects of tamoxifen on c-Myc and Bcl-2 in tamoxifen-sensitive cells to see if tamoxifen-induced CIP2A inhibition could also affect cell survival through dysregulation of other PP2A substrates that are involved in apoptosis (Additional file 1: Figure S8). Interestingly, tamoxifen showed differential effects on c-Myc and Bcl-2 in sensitive cell lines (MDA-MB-468, MDA-MB-231, MDA-MD-453 and SK-BR3). Tamoxifen reduced c-Myc expression more prominently in SK-BR3 cells and reduced Bcl-2 expression more prominently in MDA-MB-231 cells. Compared to the effects of tamoxifen on p-Akt reduction, the p-Akt downregulation was more consistent in sensitive cells and correlated better with tamoxifen-induced apoptosis (Figure 1).

In the present study, we show that tamoxifen suppressed CIP2A transcription, but did not affect the degradation of CIP2A protein after treatment with cycloheximide (Figure 3). These results suggest that tamoxifen affects CIP2A at the pretranscription level. To further decipher the possible mechanism of how tamoxifen affects CIP2A mRNA, we performed experiments with CIP2A promoter activity assay (Figure 3C), ChIP assay (Figure 3D) and Western blotting for nuclear/cytoplasmic extracts (Figure 3E). The results suggest that tamoxifen may downregulate CIP2A transcription via affecting Elk1. Further mechanistic study is needed.

The present study has some limitations. Although we show here that ERα-negative cells are sensitive to apoptosis induction by tamoxifen, it must be noted that the concentrations required to induce apoptosis in these cells is higher than for ERα-positive cells. Our *in vitro* data showed at 5 μM tamoxifen induced more apoptosis in ERα-positive MCF-7 cells compared to that in ERα-negative MDA-MB-453 cells (Additional file 1: Figure S2). A previous study by Salami *et al*. revealed a similar result in terms of apoptosis, in which MCF-7 cells responded to a lower tamoxifen concentration (1 μM) in a comparison with MDA-MB468 cells, which were mainly affected at a higher dose (20 μM) [53]. The usual effective dose of tamoxifen in humans is around 20 to 40 mg/day [26],[54]. Previous studies have shown a tumoristatic effect of tamoxifen on MCF-7 xenografted tumors in mice [55]-[58]. The effective doses of tamoxifen in these *in vivo* studies have been relatively low [55]-[58]. In addition, tamoxifen has been shown to affect cancer cell cycles at serum concentrations of tamoxifen achieved in breast cancer patients (about 1 μM) [55]. These data suggest that the doses of tamoxifen in ERα-positive xenograft mice models could inhibit cell proliferation rather than directly kill cancer cells. The dose of tamoxifen (100 mg/kg mouse body weight) used for xenografts of ERα-negative cells (MDA-MB-468) in the present study is higher than that used for xenografts of MCF-7 cells previously reported in the literature. In a pharmacokinetic study, Robinson *et al*. showed that repeated large oral doses (200 mg/kg/day for 6 days) of tamoxifen to athymic mice produced an array of serum metabolites similar to that seen in breast cancer patients [59]. Although our *in vivo* data show that tamoxifen inhibited tumor growth and downregulated protein levels of CIP2A in MDA-MB-468 xenograft tumors, we did not observe markers of apoptosis or proliferation and our results do not validate the role of CIP2A in tamoxifen-induced apoptosis *in vivo*. In this regard, more xenograft experiments using breast cancer cells with overexpression or downregulation of CIP2A and involving examination of apoptosis markers should be helpful.

Currently, the structural information of CIP2A is lacking, and the exact molecular mechanism of PP2A inhibition by CIP2A remains unclear; therefore, directly targeting CIP2A is difficult. Our data show that forskolin, a PP2A activator, sensitized HCC-1937 cells to tamoxifen-induced apoptosis (Figure 2E and Additional file 1: Figure S4). Recent studies have shown that pharmacologic restoration of PP2A tumor suppressor activity by PP2A-activating drugs (PADs; for example, forskolin, FTY720) effectively antagonizes cancer development and progression [60]. Accordingly, Perrotti *et al*. suggested the importance of PP2A as a druggable tumor suppressor in light of the possible use of PADs as anticancer agents [60].

## Conclusions

We report that tamoxifen acts through CIP2A-dependent downregulation of p-Akt-mediated, tamoxifen-induced apoptosis in ER-negative breast cancer cells. Our results support the potential of CIP2A as a therapeutic target in breast cancer treatment. Further studies designed to unravel the detailed molecular modification of CIP2A by tamoxifen and its application in other cancer cell types are warranted.

## Authors' contributions

LMT and KFC were responsible for coordination and manuscript editing as well as acting as corresponding authors. CYL drafted the manuscript. CYL, MHH, DSW, PYC, JCS, THT, CTH, TTC, and CYW conducted *in vitro* experiments. CYL, MHH, DSW, PYC, JCS, THT, and CTH conducted animal and histopathological experiments. CYL, MHH, CTH, TTC, CYW, LMT, and KFC performed or helped clinical data acquisition and analysis. CWS, LMT, and KFC helped in data interpretation and statistical analysis. CYL, MHH, DSW, PYC, JCS, THT, CTH, TTC, CYW and CWS prepared the figures. All authors had substantial contributions to the conception or design of the work. All authors read the final manuscript, revised it critically for intellectual content and approved the final manuscript. All authors agreed with the accuracy and integrity of all parts of the work.

## Additional file

## Electronic supplementary material

Additional file 1: Figure S1.: Effect of tamoxifen in MCF-7 cells. Cells were exposed to tamoxifen at the indicated doses for 36 hours. **Figure S2.** Effects of tamoxifen and fulvestrant on ERα and CIP2A. Cells were treated with these agents at indicated doses for 36 hours. Fulvestrant was purchased from Sigma-Aldrich (St Louis, MO, USA). **Figure S3.** Expression of ERα in MCF-7 and ERα-negative breast cancer cells. **Figure S4.** Cotreatment of tamoxifen with forskolin enhanced PP2A activity in resistant HCC-1937 cells. Cells were treated with DMSO or tamoxifen (7.5 μM) or cotreated with tamoxifen (7.5 μM) and forskolin (40 μM) for 36 hours. Columns, mean values (*n* = 3); bars, SD; *P < 0.05. **Figure S5.** Downregulation of CIP2A by siRNA increased tamoxifen-induced apoptosis in MDA-MB-468 cells. Cells were transfected with either scrambled or CIP2A siRNA for 72 hours, followed by exposure to tamoxifen for 36 hours. Columns, mean values (*n* = 3); bars, SD; *P < 0.05. **Figure S6.** Effects of tamoxifen and common chemotherapeutic agents on apoptosis associated with CIP2A expression. Cells were treated with DMSO, tamoxifen (5 μM), 5-FU (40 μM), paclitaxel (20 nM) or docetaxel (2 μM) for 36 hours and assayed for CIP2A and apoptosis. **Figure S7.** Coimmunoprecipitation of CIP2A and Hsp90 in MDA-MB-468 cells treated with or without tamoxifen for 36 hours. **Figure S8.** Effects of tamoxifen on c-Myc and Bcl-2 expressions in tamoxifen-sensitive ERα-negative breast cancer cells. Cells were treated with DMSO or tamoxifen for 36 hours. (PPTX 489 KB)

Below are the links to the authors’ original submitted files for images.

Authors’ original file for figure 1

Authors’ original file for figure 2

Authors’ original file for figure 3

Authors’ original file for figure 4

Authors’ original file for figure 5

Authors’ original file for figure 6

## Abbreviations

CIP2A: Cancerous inhibitor of protein phosphatase 2A
DMSO: Dimethyl sulfoxide
ER: Estrogen receptor
HER2: Human epidermal growth factor receptor type 2
Hsp: Heat shock protein
PARP: Poly(ADP-ribose) polymerase
PP2A: Protein phosphatase 2A
siRNA: Small interfering RNA
