# Supplementary figures and images for: Tamoxifen induces apoptosis through cancerous inhibitor of protein phosphatase 2A–dependent phospho-Akt inactivation in estrogen receptor–negative human breast cancer cells
Source: Breast Cancer Res. 2014 Sep 17;16:431. doi: 10.1186/s13058-014-0431-9 (PMC4303112; doi:10.1186/s13058-014-0431-9)

## Slide 1
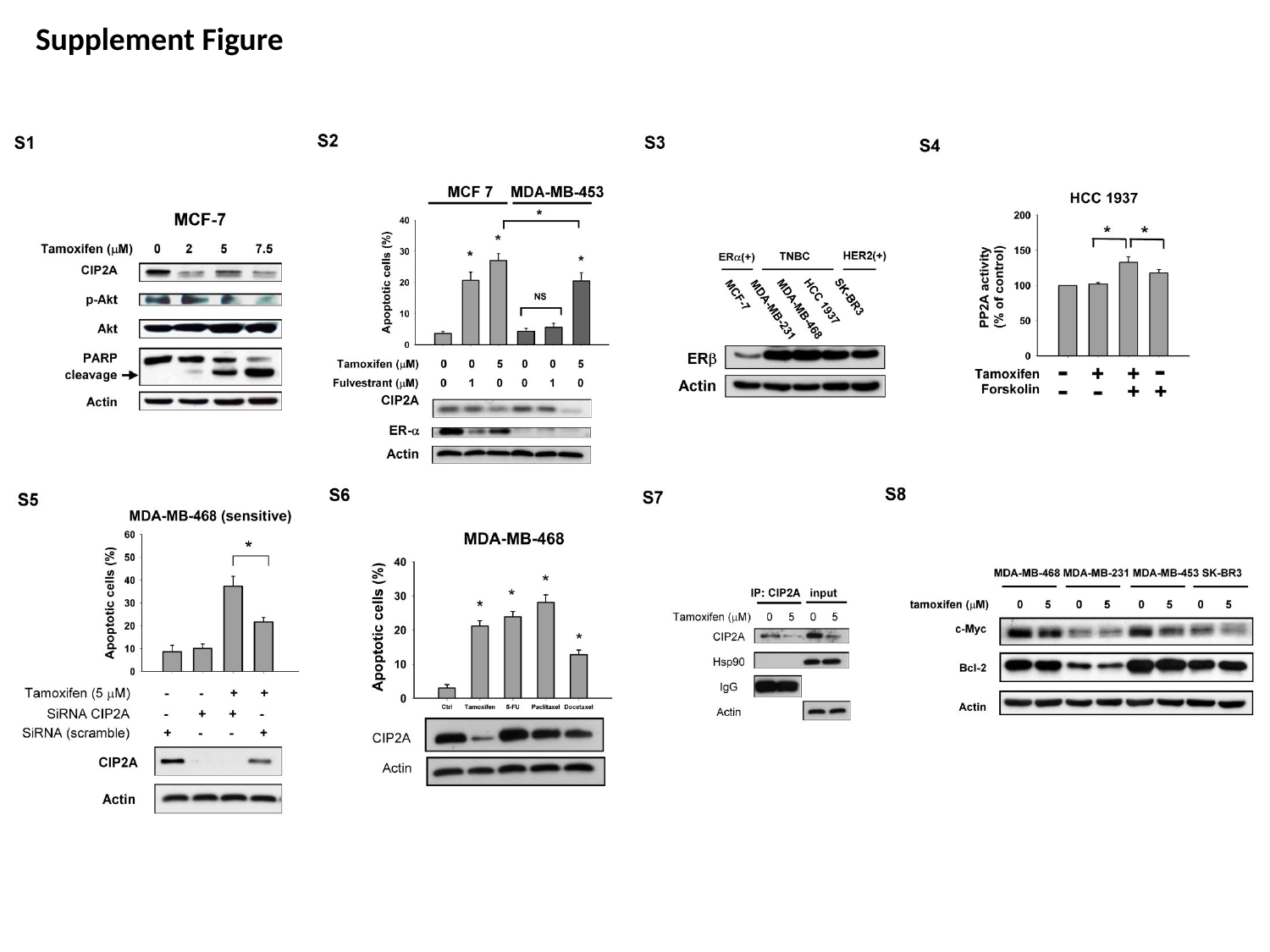

Supplement Figure

Supplement: Supplementary file 1 — Additional file 1: Figure S1.: Effect of tamoxifen in MCF-7 cells. Cells were exposed to tamoxifen at the indicated doses for 36 hours. Figure S2. Effects of tamoxifen and fulvestrant on ERα and CIP2A. Cells were treated with these agents at indicated doses for 36 hours. Fulvestrant was purchased from Sigma-Aldrich (St Louis, MO, USA). Figure S3. Expression of ERα in MCF-7 and ERα-negative breast cancer cells. Figure S4. Cotreatment of tamoxifen with forskolin enhanced PP2A activity in resistant HCC-1937 cells. Cells were treated with DMSO or tamoxifen (7.5 μM) or cotreated with tamoxifen (7.5 μM) and forskolin (40 μM) for 36 hours. Columns, mean values (n = 3); bars, SD; *P < 0.05. Figure S5. Downregulation of CIP2A by siRNA increased tamoxifen-induced apoptosis in MDA-MB-468 cells. Cells were transfected with either scrambled or CIP2A siRNA for 72 hours, followed by exposure to tamoxifen for 36 hours. Columns, mean values (n = 3); bars, SD; *P < 0.05. Figure S6. Effects of tamoxifen and common chemotherapeutic agents on apoptosis associated with CIP2A expression. Cells were treated with DMSO, tamoxifen (5 μM), 5-FU (40 μM), paclitaxel (20 nM) or docetaxel (2 μM) for 36 hours and assayed for CIP2A and apoptosis. Figure S7. Coimmunoprecipitation of CIP2A and Hsp90 in MDA-MB-468 cells treated with or without tamoxifen for 36 hours. Figure S8. Effects of tamoxifen on c-Myc and Bcl-2 expressions in tamoxifen-sensitive ERα-negative breast cancer cells. Cells were treated with DMSO or tamoxifen for 36 hours. (PPTX 489 KB) [file 13058_2014_431_MOESM1_ESM.pptx]

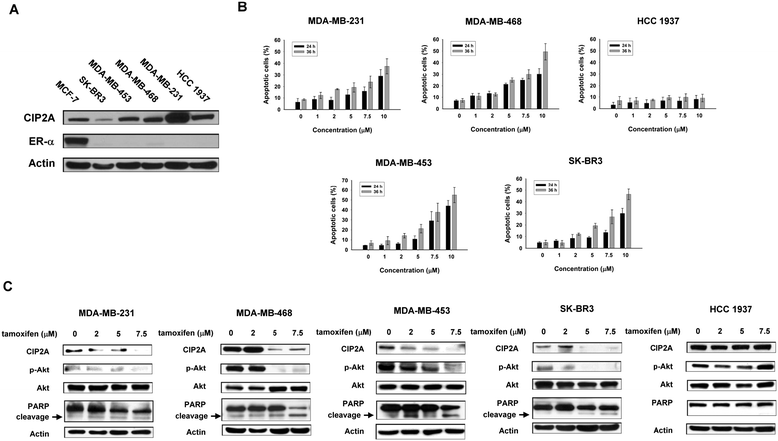

Supplement: Supplementary file 2 — Authors’ original file for figure 1 [file 13058_2014_431_MOESM2_ESM.gif]

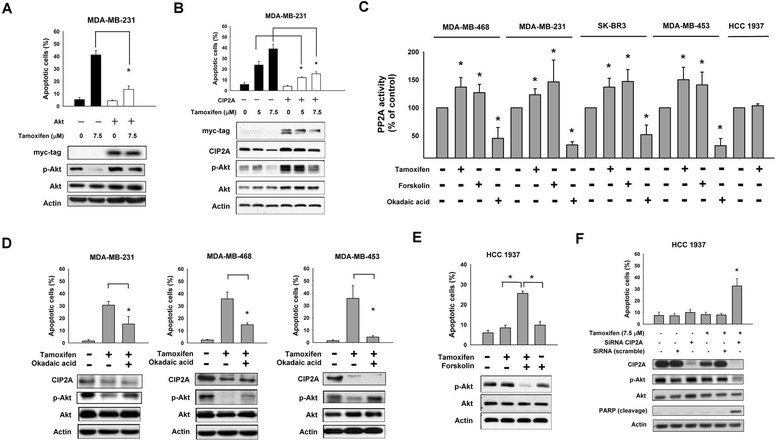

Supplement: Supplementary file 3 — Authors’ original file for figure 2 [file 13058_2014_431_MOESM3_ESM.gif]

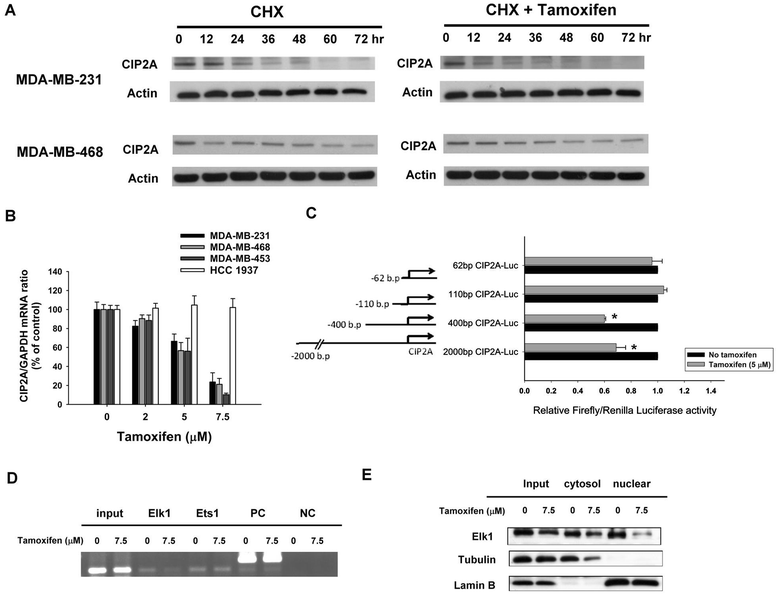

Supplement: Supplementary file 4 — Authors’ original file for figure 3 [file 13058_2014_431_MOESM4_ESM.gif]

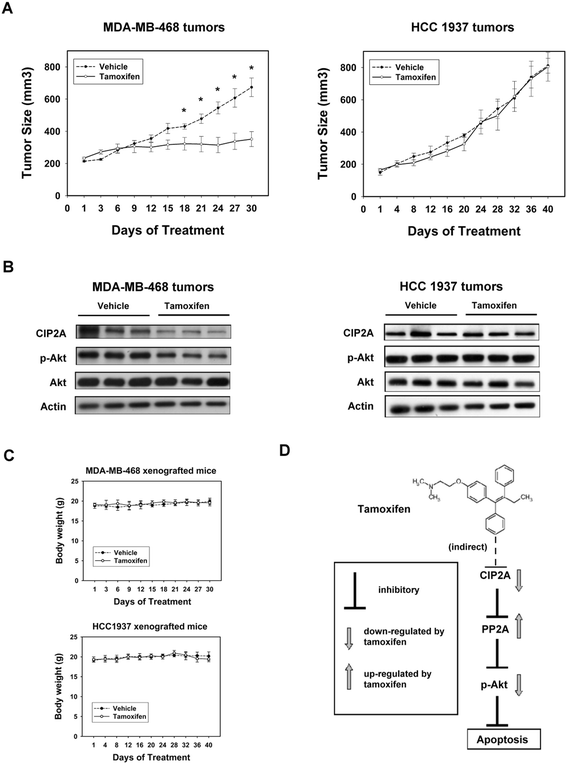

Supplement: Supplementary file 5 — Authors’ original file for figure 4 [file 13058_2014_431_MOESM5_ESM.gif]

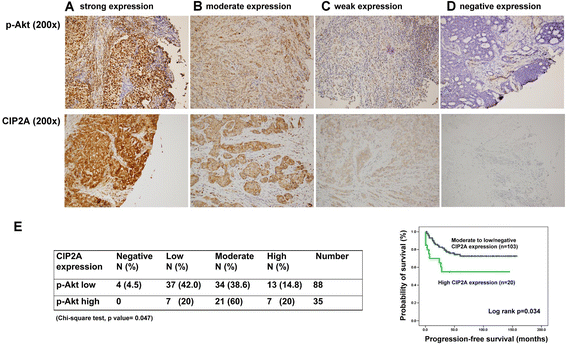

Supplement: Supplementary file 6 — Authors’ original file for figure 5 [file 13058_2014_431_MOESM6_ESM.gif]
